# Integrated approaches to geriatric mental disorders: pathophysiology, interventions, and precision medicine horizons

**DOI:** 10.3389/fpsyt.2026.1946385

**Published:** 2026-09-04

**Authors:** Jin Wang, Anqi Li

**Affiliations:** Lanzhou First People’s Hospital, Lanzhou, Gansu, China

**Keywords:** collaborative care, geriatric mental disorders, late-life depression, neuroinflammation, pharmacogenomics, precision medicine

## Abstract

The global aging demographic has precipitated a surge in late-life mental disorders (LMDs)—including late-life depression (LLD), anxiety, and behavioral and psychological symptoms of dementia (BPSD)—posing critical public health challenges. This review synthesizes current evidence on the unique pathophysiology, clinical management, and emerging frontiers of geriatric mental health, emphasizing the interplay of neurobiological aging, multimorbidity, and psychosocial stressors. Key pathophysiological mechanisms include “inflammaging” (microglial overactivation), cerebral small vessel disease (CSVD), and senescence of monoaminergic systems, which collectively reduce neural resilience and complicate diagnosis. Clinical challenges—such as atypical symptom presentation (e.g., somatic masking of depression), polypharmacy risks, and diagnostic ambiguity between LLD and pseudodementia—necessitate tailored interventions. Notably, caregiver burden represents an independent predictor of patient institutionalization. Multiple systematic reviews and meta-analyses have consistently identified caregiver burden and distress as significant predictors of nursing home admission, with carer stress sometimes serving as the single determining factor for this transition. This underscores the critical need to integrate caregiver well-being into routine clinical assessment. Pharmacological strategies prioritize SSRIs/SNRIs but require cautious dosing due to age-related pharmacokinetic alterations (e.g., reduced renal clearance) and cardiovascular/metabolic risks. Emerging targets (e.g., ketamine for treatment-resistant LLD, anti-inflammatory agents guided by biomarker profiling) highlight a shift toward precision. Psychotherapeutic adaptations—including modified cognitive behavioral therapy (CBT), problem-solving therapy (PST), and life review therapy—address cognitive and psychosocial needs, while integrated care models (e.g., Collaborative Care Model [CoCM]) demonstrate superior outcomes over fragmented care. Lifestyle interventions (exercise) and neuromodulation (rTMS) further expand treatment options. Critical gaps remain, including underrepresentation of older adults in clinical trials and pervasive polypharmacy. Future directions emphasize pharmacogenomics, AI-driven early detection, and culturally equitable care to advance precision psychiatry for LMDs. This synthesis provides clinicians and researchers with a roadmap to navigate the complexities of geriatric mental health, advocating for multidisciplinary, patient-centered approaches to improve outcomes in aging populations.

## 1 Introduction

### 1.1 Epidemiological landscape: the growing burden of geriatric mental disorders

The global demographic shift towards an aging population has precipitated a concurrent rise in the prevalence of late-life mental disorders, positioning them as a critical public health priority. LLD is among the most prevalent psychiatric conditions in the elderly.

According to the latest Global Burden of Disease Study 2021, the global age-standardized incidence rate of LLD remains persistently high, with hundreds of millions of new cases emerging annually, contributing substantially to disability-adjusted life years (DALYs) in individuals over 65 (1). Critically, the clinical presentation of LLD often diverges from that of younger adults, frequently manifesting as apathy, cognitive slowing, or somatic complaints rather than overt low mood. Recent comprehensive syntheses emphasize that extrapolating diagnostic criteria validated in younger populations to geriatric cohorts risks significant underdiagnosis or misclassification, necessitating age-specific assessment frameworks (2). Concurrently, anxiety disorders pose a significant threat to elderly mental well-being; a recent large-scale systematic review and meta-analysis estimated the global pooled prevalence of anxiety symptoms in older adults to be approximately 16.5%, though rates can vary significantly based on regional and diagnostic methodologies (3).

Furthermore, as the aging process accelerates neurodegenerative pathways, a substantial proportion of the elderly population grapples with dementia and its associated neuropsychiatric sequelae. Behavioral and Psychological Symptoms of Dementia (BPSD)—also referred to as Neuropsychiatric Symptoms (NPS)—encompass a wide array of manifestations including apathy, agitation, depression, and psychosis. Meta-analytic evidence suggests that the overall prevalence of BPSD in dementia patients is strikingly high, affecting up to 75.7% of cases, with severe ramifications for patient quality of life and caregiver burden (1, 4). Notably, BPSD are not confined to advanced dementia; milder forms, termed Mild Behavioral Impairment (MBI), often presage cognitive decline, underscoring the continuum of neuropsychiatric dysfunction in late life (5). Collectively, these disorders impose an immense socioeconomic toll.

### 1.2 Clinical challenges: diagnostic complexity and therapeutic hurdles

Despite the clear epidemiological imperative, the clinical management of mental disorders in the elderly is fraught with unique complexities. A primary challenge lies in the exceptionally high rate of multimorbidity. Older adults frequently contend with a constellation of chronic physical ailments—such as cardiovascular disease, diabetes, and chronic pain—which not only exacerbate psychiatric symptoms but also complicate pharmacological treatment due to polypharmacy and altered pharmacokinetics (6).

Moreover, the phenomenology of mental illness in late life is often atypical, leading to profound diagnostic obfuscation. Elderly patients with depression frequently exhibit a predominance of somatic complaints and a notable paucity of overt sadness. This clinical presentation, often rooted in age-related psychological coping mechanisms such as alexithymia (the inability to identify and describe one’s own emotions), results in the “masking” of underlying depression (7). Conversely, the cognitive deficits and psychomotor retardation inherent in severe LLD can closely mimic neurodegenerative dementia, a phenomenon classically described as “pseudodementia” (8). Distinguishing between primary psychiatric distress, somatic manifestation, and incipient dementia requires nuanced clinical acumen, which is often hindered by the patient’s reduced insight and reluctance to engage in traditional psychiatric discourse. These diagnostic ambiguities, coupled with ageist misconceptions that dismiss psychiatric symptoms as an inevitable consequence of aging, invariably lead to delayed interventions and suboptimal therapeutic outcomes.

### 1.3 Search strategy and selection criteria

This narrative review synthesizes current evidence regarding the pathophysiology, clinical management, and horizons of precision psychiatry​ in geriatric mental disorders. We searched four electronic databases—PubMed, Web of Science Core Collection, Cochrane Library, and PsycINFO—from January 1990 to May 2026, using keyword combinations spanning four core domains: (1) population: “older adults”, “elderly”, “geriatric”; (2) conditions: “LLD”, “anxiety disorders”, “behavioral and psychological symptoms of dementia”, “BPSD”; (3) mechanisms: “inflammaging”, “cerebral small vessel disease”, “CSVD”, “neuroinflammation”, “HPA axis”; (4) interventions: “antidepressants”, “collaborative care”, “pharmacogenomics”, “artificial intelligence”. We included peer-reviewed original studies, systematic reviews, and meta-analyses focused on populations aged ≥65 years, prioritizing publications from the past 5 years alongside seminal earlier works (e.g., the IMPACT trial). Exclusion criteria comprised non-English publications, case reports, conference abstracts, and studies exclusively enrolling non-geriatric participants. Literature selection was thematic, targeting studies with high clinical or mechanistic relevance to the review’s core framework of pathophysiology, intervention, and biomarker-guided therapeutic strategies, to ensure balanced coverage of multidisciplinary perspectives.

### 1.4 Objectives and scope of the review

In light of these multifaceted challenges, this review aims to provide a comprehensive synthesis of the current landscape of geriatric mental health, with a specialized focus on LLD, anxiety, and BPSD. The primary objectives are threefold: First, to elucidate the unique pathophysiological trajectories of these disorders in the aging brain, integrating insights from neuroinflammation, vascular pathology, and neurotransmitter dynamics. Second, to critically evaluate contemporary intervention strategies, juxtaposing the efficacy, safety profiles, and limitations of conventional pharmacotherapies against emerging psychotherapeutic modalities tailored for the elderly. Finally, this review seeks to pave the way for precision medicine in geriatric psychiatry by identifying critical gaps in the current literature and proposing a framework for integrated, multidisciplinary care. Through this comprehensive analysis, we aim to equip clinicians and researchers with the requisite knowledge to navigate the intricacies of late-life mental disorders and ultimately improve the trajectory of mental health aging.

## 2 Unique pathophysiology of geriatric mental disorders

**Figure 1:**
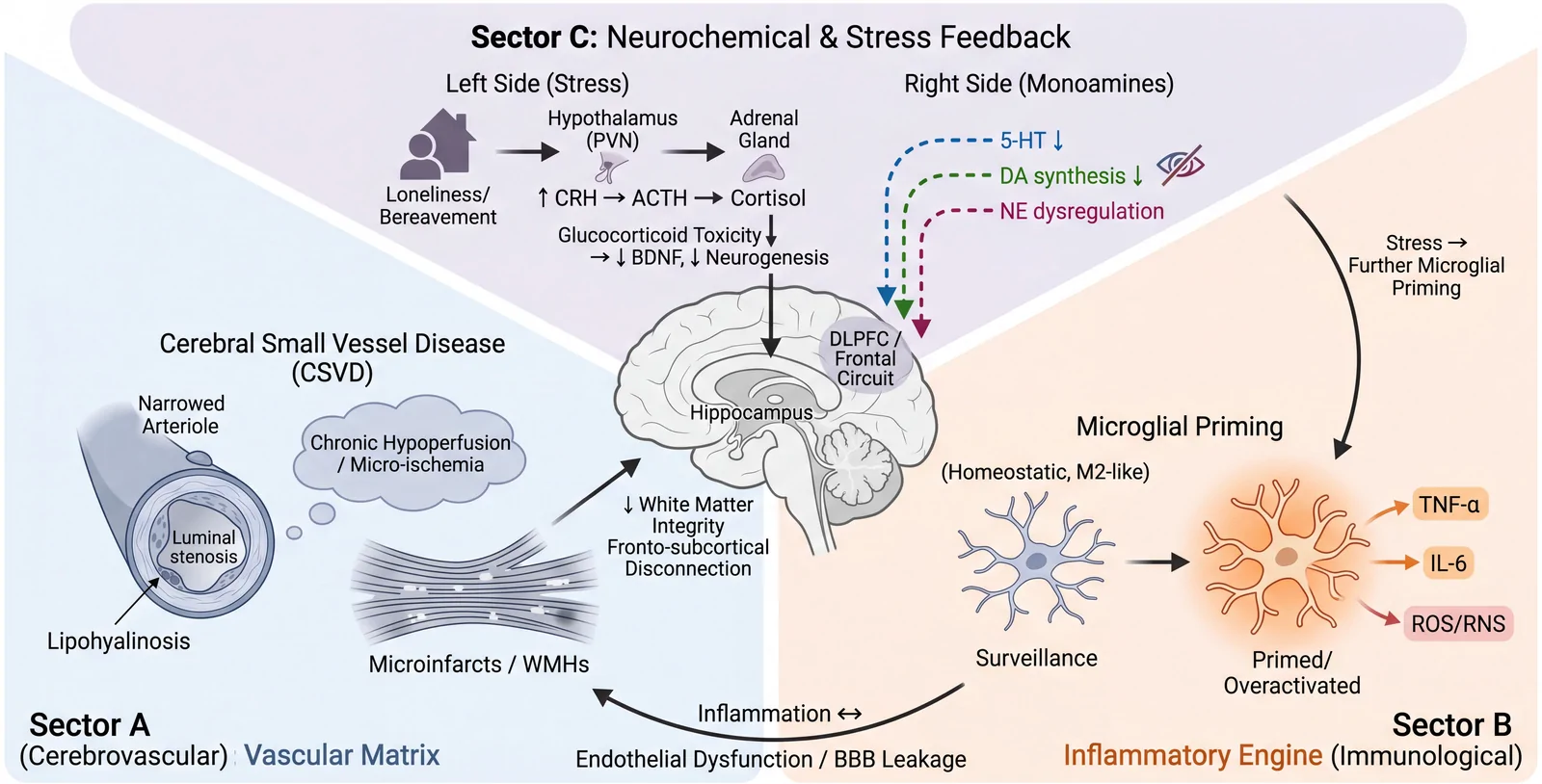
The vicious cycle of geriatric mental pathology.

The pathophysiology of mental disorders in late life is fundamentally distinct from that of younger populations. Rather than stemming from a single monoaminergic deficit, geriatric mental disorders arise from a complex interplay of age-related neurobiological changes—including neuroinflammation, cerebrovascular pathology, and neurotransmitter system senescence—compounded by profound psychosocial stressors. Understanding these unique mechanistic substrates is critical for developing targeted interventions, as shown in **Figure 1**. **Figure 1** illustrates this convergence, depicting how neurobiological aging (inflammaging and CSVD), neurotransmitter system senescence, and psychosocial stressors interact synergistically. Crucially, the overlap of these domains—rather than any single factor—reduces neural resilience and precipitates the transition from healthy aging to clinical disorder.

### 2.1 Neurobiological alterations

#### 2.1.1 Neuroinflammation and oxidative stress: the role of microglial overactivation

A hallmark of the aging brain is a chronic, low-grade neuroinflammatory state, often referred to as “inflammaging.” Central to this process is the overactivation of microglia, the brain’s resident innate immune cells. Under homeostatic conditions, microglia exhibit a neuroprotective phenotype (M2), supporting synaptic plasticity and clearing cellular debris. However, with advancing age, microglia undergo a phenotypic shift toward a pro-inflammatory (M1) state (9).

This age-related priming makes microglia hyper-responsive to peripheral insults (e.g., infection or systemic illness). Upon activation, these primed microglia release excessive pro-inflammatory cytokines (e.g., TNF-α, IL-6) and chemokines, which can precipitate or exacerbate depressive symptoms and accelerate neurodegenerative processes (9). Mechanistically, neuroinflammation disrupts monoaminergic neurotransmission by inducing the enzyme indoleamine 2,3-dioxygenase (IDO), which shunts tryptophan metabolism away from serotonin synthesis toward the production of neurotoxic kynurenine metabolites (10, 11). Furthermore, recent evidence indicates that microglial overactivation promotes the release of amyloid-beta (Aβ)-containing exosomes via lactate-dependent modulation of potassium channels (Kv1.3), thereby accelerating the spread of AD pathology (12). Clinical Translation:​ Identifying an “inflammatory phenotype”—typically defined by elevated peripheral biomarkers—directly informs therapeutic strategy. Rather than solely escalating antidepressant doses, clinicians may consider adjuvant anti-inflammatory therapies (e.g., celecoxib) or targeted lifestyle interventions (e.g., omega-3 supplementation, aerobic exercise) for these patients. Routine inflammatory biomarker screening thus offers a pragmatic pathway to personalize treatment selection in LLD.

#### 2.1.2 Vascular contributions: CSVD

Vascular pathology is inextricably linked to LLD and dementia, often acting as a shared underlying mechanism. CSVD, driven by arteriolosclerosis and cerebral amyloid angiopathy (CAA), is highly prevalent in the elderly and frequently coexists with neurodegenerative pathologies (13, 14).

CSVD disrupts the blood-brain barrier (BBB), impairs cerebral blood flow autoregulation, and leads to chronic hypoperfusion and hypoxia (14, 15). These microvascular lesions manifest neuroradiologically as white matter hyperintensities (WMHs), lacunes, and microbleeds (14, 16). The resulting damage to fronto-subcortical circuits is a key pathophysiological substrate of “vascular depression,” characterized by executive dysfunction, psychomotor retardation, and apathy (16, 17). Moreover, CSVD synergizes with Alzheimer’s disease pathology; BBB breakdown and interstitial fluid drainage failure promote the accumulation of Aβ and tau proteins, significantly increasing the risk of mixed dementia (13, 15). Clinical Translation:​ The presence of WMHs and lacunes on neuroimaging identifies a “vascular depression” subtype characterized by executive dysfunction and poor antidepressant response. Clinically, this necessitates aggressive management of vascular risk factors (e.g., strict BP control, statins) alongside pharmacotherapy. Recognizing CSVD burden also aids prognosis, as extensive white matter damage predicts a higher likelihood of progression from mild cognitive impairment to dementia.

#### 2.1.3 Neuroimaging correlates of CSVD and atrophy

The structural sequelae of inflammaging and CSVD are readily quantifiable via modern neuroimaging, bridging pathological processes to clinical phenotypes. Computed Tomography (CT) remains valuable in acute settings to exclude hemorrhage or large infarcts, yet Magnetic Resonance Imaging (MRI) is the gold standard for characterizing CSVD burden. Key radiological markers—including White Matter Hyperintensities (WMHs), lacunes, and microbleeds—correlate strongly with executive dysfunction and the “vascular depression” phenotype (18, 19). Beyond vascular lesions, structural MRI allows for the quantification of regional brain atrophy. Notably, hippocampal volume loss aids in differentiating LLD from incipient neurodegenerative dementia (pseudodementia), as LLD typically presents with less medial temporal lobe atrophy compared to Alzheimer’s disease (20–22). Thus, routine neuroimaging serves as a critical tool to deconstruct the heterogeneity of geriatric mental disorders.

#### 2.1.4 Senescence of neurotransmitter systems

Aging is accompanied by the progressive degeneration of monoaminergic neurotransmitter systems, which fundamentally alters neural signaling and emotional regulation.

Dopaminergic Decline:​ Dopamine synthesis in the striatum and prefrontal cortex declines significantly with age, accompanied by a marked loss of D1, D2, and D3 receptors (23). This dopaminergic denervation contributes to the psychomotor slowing, executive deficits, and anhedonia commonly observed in LLD (23).

Serotonergic Alterations:​ Aging leads to a reduction in the density of serotonergic projections and a downregulation of 5-HT receptors (particularly 5-HT2) in the frontal cortex and hippocampus (23). The consequent disruption of serotonin homeostasis impairs emotional processing and sleep architecture (23).

Noradrenergic Imbalance:​ While baseline norepinephrine levels may appear stable, the aging locus coeruleus experiences a loss of noradrenergic cell bodies. This, combined with altered receptor sensitivity, disrupts the stress response and attentional control mechanisms (23, 24).

Crucially, the structural and functional degradation of these neurotransmitter systems reduces the brain’s resilience to stress and limits the efficacy of traditional pharmacotherapies that rely on synaptic plasticity (24). Clinical Translation:​ The specific pattern of neurotransmitter decline guides symptom-targeted prescribing. For instance, prominent psychomotor retardation or anhedonia—reflecting dopaminergic denervation—may warrant augmentation with bupropion or high-frequency rTMS rather than standard SSRI monotherapy. Conversely, understanding serotonergic downregulation supports cautious SSRI dosing to avoid adverse events (e.g., hyponatremia) in this sensitive population.

### 2.2 Psychosocial factors and the HPA axis

Beyond neurobiology, profound psychosocial transitions in late life act as potent stressors that reshape the brain’s endocrine environment. Events such as retirement, bereavement, and the onset of chronic physical illnesses often lead to social isolation and loneliness.

Social isolation and perceived loneliness function as chronic psychological stressors that profoundly dysregulate the Hypothalamic-Pituitary-Adrenal (HPA) axis (25, 26). Meta-analytic evidence highlights that loneliness is significantly associated with alterations in diurnal cortisol rhythms, including a blunted cortisol awakening response and diminished overall cortisol output in older adults (25, 27). While counterintuitive compared to the hypercortisolemia seen in acute stress, this flattened cortisol profile reflects a state of HPA axis exhaustion or sensitization (27).

Chronic HPA axis dysregulation exacerbates neuroinflammation, impairs hippocampal neurogenesis, and accelerates telomere shortening, thereby creating a vicious cycle that heightens vulnerability to both depression and cognitive decline (26). This neuroendocrine-immune interface is further characterized by altered cellular immune sensitivity to cortisol. Longitudinal studies in Alzheimer’s disease (AD) indicate that natural killer (NK) cell cytotoxicity becomes hyper-responsive to physiological cortisol concentrations, with this dysregulation correlating with faster disease progression (28). Moreover, dynamic interactions between cortisol-modulated NK activity and circulating activated T-lymphocytes reveal a significant negative correlation, particularly pronounced in rapidly progressing AD subtypes (29). These findings substantiate a mechanistic link between HPA axis hyperactivity, cellular immune senescence, and the pathogenesis of geriatric neuropsychiatric disorders. Furthermore, the lack of social integration deprives the elderly of the “social buffering” effect—a neurobiological phenomenon wherein positive social interactions mitigate stress-induced HPA axis activation. Consequently, the combination of age-related neurobiological fragility and psychosocial stress creates a perfect storm for the onset and perpetuation of geriatric mental disorders. Clinical Translation:​ A blunted cortisol awakening response or reported social isolation should prompt clinicians to prioritize psychosocial interventions over pharmacological escalation. Integrating Problem-Solving Therapy (PST) or Life Review Therapy (LRT) can restore stress resilience by mitigating HPA axis dysregulation. This bidirectional stress dynamic extends to caregivers: chronic cortisol elevation in overwhelmed caregivers can be transmitted to older adults via daily interpersonal interactions, exacerbating the patient’s HPA axis dysregulation and worsening psychiatric symptoms. Furthermore, assessing caregiver support and social networks is essential, as enhancing “social buffering” directly counters neuroendocrine vulnerability and improves long-term outcomes.1-5

### 2.3 Translational implications: from pathophysiological subtypes to targeted interventions

This pathophysiological heterogeneity—spanning microglial activation, cerebrovascular lesions, and neurotransmitter senescence—necessitates a departure from the unidimensional monoaminergic deficiency model that dominates younger-adult psychiatric care. Rather than relying on symptom-based diagnosis alone, clinicians require objective biomarkers to guide personalized decision-making, and neuroimaging has emerged as a pivotal translational tool to meet this need. As visualized in **Figure 1**, late-life mental disorders arise not from a single pathological driver, but from a confluence of neuroinflammation, vascular pathology, neurotransmitter system senescence, and neuroendocrine dysregulation—a multisystem interplay that neuroimaging can now partially quantify to inform targeted intervention.

This mechanistic heterogeneity implies that a uniform, symptom-based treatment algorithm is insufficient for the aged brain.

Translating these findings into clinical practice necessitates a shift toward mechanism-guided precision care. For instance, the identification of an “inflammatory phenotype”—characterized by elevated peripheral biomarkers (e.g., CRP, IL-6)—opens avenues for adjuvant anti-inflammatory therapies or specific lifestyle interventions targeting systemic inflammation, rather than merely escalating antidepressant doses. Similarly, recognizing CSVD as a driver of executive dysfunction and “vascular depression” mandates the integration of vascular risk factor management with psychiatric treatment. In contrast, patients exhibiting predominant dopaminergic decline may require interventions specifically targeting psychomotor retardation and anhedonia, such as dopaminergic augmentation or high-frequency rTMS.

Furthermore, the profound impact of HPA axis dysregulation​ and psychosocial stressors (e.g., loneliness) underscores the necessity of multimodal interventions. While pharmacotherapy addresses neurobiological deficits, psychotherapeutic adaptations (e.g., Problem-Solving Therapy, Life Review) are essential to restore stress resilience and counteract the deleterious effects of social isolation. **Table 1** synthesizes these correspondences, providing a roadmap for aligning biological substrates with therapeutic strategies, thereby setting the stage for the integrated management approaches detailed in the following sections.

**Table 1:** Mechanistic substrates and corresponding targeted interventions in geriatric mental disorders.

Pathophysiological driver	Key biomarkers/clinical features	Targeted intervention strategy	Expected clinical benefit
Neuroinflammation (§2.1.1)	• ↑ CRP, IL-6, TNF-α• Microglial overactivation (M1 phenotype)• Tryptophan depletion	Pharmacological:• COX-2 inhibitors (Celecoxib)*• Cytokine inhibitors (Infliximab)* Lifestyle:• Omega-3 fatty acids• Aerobic exercise (↑ BDNF)	• Faster remission in inflammatory phenotype• Reduced neuroprogression• Improved treatment resistance
Cerebrovascular Pathology (CSVD) (§2.1.2)	• White matter hyperintensities (WMHs)• Lacunes, microbleeds• Executive dysfunction, apathy	Medical Management:• Strict BP control (<130/80 mmHg)• Antiplatelet therapy (as indicated)• Lipid-lowering agents Psychiatric: SNRIs (for vascular depression)	• Slowed progression to dementia• Improved executive function• Reduced apathy severity
Neurotransmitter Senescence (§2.1.3)	• Striatal dopamine decline• ↓ 5-HT_2_ receptor density• Locus coeruleus atrophy	Pharmacological:• Low-dose SSRIs/SNRIs (cautious PK)• Bupropion (for anhedonia) Neuromodulation:* • High-frequency rTMS (DLPFC)	• Restoration of emotional processing• Reduced psychomotor slowing• Enhanced synaptic plasticity
HPA Axis & Psychosocial Stress (§2.2)	• ;Blunted cortisol awakening response• Social isolation, loneliness• Alexithymia	Psychotherapeutic:• Problem-Solving Therapy (PST)• Life Review Therapy (LRT)• Behavioral Activation (BA) Social:• Caregiver support• Community integration	• Restored stress resilience• Decreased caregiver burden• Improved existential well-being

Indicates off-label use or agents requiring careful risk-benefit assessment in the elderly. ↑: Increase / elevation; ↓: Decrease / reduction; §: Cross-reference to a specific section; *: Off-label use or requires careful risk-benefit assessment.

**Table 1**. Mechanistic substrates and corresponding targeted interventions in geriatric mental disorders.

## 3 Pharmacological interventions

The selection of pharmacotherapies in late life must be mechanistically informed. As summarized in **Table 1**, interventions should target specific pathophysiological drivers—ranging from neurotransmitter senescence to neuroinflammation—rather than solely addressing symptomatic presentations. While psychotherapeutic modalities remain indispensable in the management of geriatric mental disorders, pharmacological interventions often serve as the frontline treatment, particularly in moderate-to-severe cases or when cognitive impairments hinder psychotherapy adherence. However, prescribing psychotropics in late life is intrinsically complex, dictated by age-related physiological changes, heightened sensitivity to adverse effects, and a precarious balancing act between therapeutic efficacy and safety.

### 3.1 Antidepressant medications

Building on the concept of neurotransmitter senescence and altered pharmacokinetics outlined in **Table 1** and Section 2.1.3, antidepressant selection in late life prioritizes safety profiles compatible with age-related biological decline. Antidepressants are the cornerstone of LLD treatment. Over the past decades, the prescription landscape has dramatically shifted from Tricyclic Antidepressants (TCAs)—owing to their prohibitive anticholinergic and cardiovascular toxicity—towards Selective Serotonin Reuptake Inhibitors (SSRIs) and Serotonin-Norepinephrine Reuptake Inhibitors (SNRIs) (30–33).

#### 3.1.1 SSRIs vs. SNRIs: efficacy, cardiovascular safety, and hemodynamic risks

SSRIs (e.g., sertraline, escitalopram) and SNRIs (e.g., venlafaxine, duloxetine) are generally equipotent in alleviating geriatric depressive symptoms. However, their side-effect profiles necessitate distinct clinical considerations, particularly concerning cardiovascular safety—a paramount concern given the high prevalence of comorbid cardiovascular disease (CVD) in the elderly (30, 31).

SSRIs are frequently criticized for their potential to induce hyponatremia via the Syndrome of Inappropriate Antidiuretic Hormone Secretion (SIADH), a serious adverse event occurring in 10–15% of older adults that can precipitate falls and confusion. Furthermore, certain SSRIs pose specific cardiac risks: citalopram and its active enantiomer escitalopram are notorious for dose-dependent QTc interval prolongation, necessitating strict dosage caps (≤20 mg/day for citalopram) in the elderly to avert malignant arrhythmias (30, 31).

Conversely, SNRIs are generally associated with a lower risk of hyponatremia but present unique hemodynamic challenges. Venlafaxine and duloxetine can stimulate noradrenergic receptors, potentially leading to elevated blood pressure, tachycardia, and palpitations. Additionally, both SSRIs and SNRIs inhibit platelet aggregation by depleting platelet serotonin stores, thereby amplifying the risk of gastrointestinal or intracranial bleeding, especially when co-administered with NSAIDs or anticoagulants like warfarin (30, 31). Therefore, meticulous cardiovascular risk stratification and judicious drug selection are imperative.

#### 3.1.2 Age-related pharmacokinetic alterations

**Figure 2:**
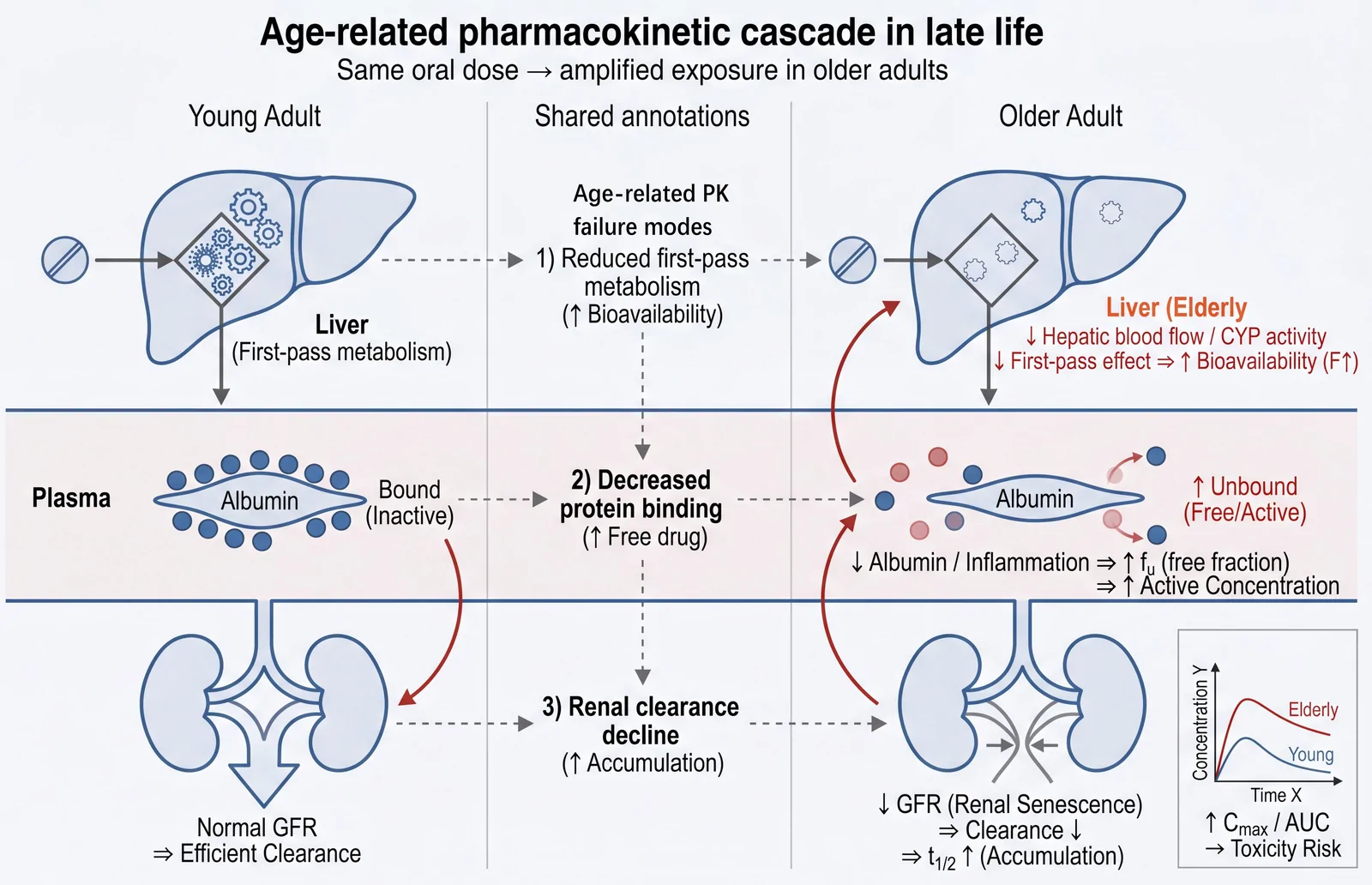
Altered pharmacokinetics in the aging body. Physiological aging amplifies drug exposure following a standard oral dose through three interrelated mechanisms: (1) Reduced hepatic clearance—declined hepatic blood flow and CYP450 activity diminish the first-pass effect, increasing bioavailability (F); (2) Decreased plasma protein binding—chronic inflammation and reduced albumin elevate the free fraction (f_u_) of pharmacologically active drug; and (3) Declining renal function—reduced glomerular filtration rate (GFR) compromises clearance of water-soluble agents, extending half-life (t_1/2_). The inset illustrates that these combined alterations result in elevated maximum plasma concentration (C_max_) and area under the plasma concentration-time curve (AUC) in older adults, significantly increasing the risk of toxic delirium and cardiac complications.

The maxim “start low, go slow” is not merely a clinical adage but a physiological necessity in geriatric pharmacotherapy, grounded in profound PK/PD shifts. Firstly, the age-related decline in hepatic blood flow and microsomal enzyme activity diminishes the “first-pass effect,” resulting in elevated bioavailability of drugs like tricyclics and certain SSRIs. Secondly, chronic inflammation and malnutrition common in old age alter plasma protein binding (e.g., decreased albumin), leading to a higher fraction of unbound, pharmacologically active drug circulating in the bloodstream, as shown in **Figure 2**. The triad of reduced hepatic clearance, decreased plasma protein binding, and declining renal function creates a ‘perfect storm’ for drug accumulation. This pharmacokinetic profile mandates aggressive dose reductions to prevent toxic delirium, particularly for water-soluble agents such as citalopram or venlafaxine.

Finally, the senescence of renal function—characterized by a natural decline in Glomerular Filtration Rate (GFR)—severely compromises the clearance of water-soluble antidepressants (e.g., citalopram, sertraline, venlafaxine). This triad of physiological changes culminates in drastically reduced drug clearance, prolonged half-lives, and drug accumulation, mandating aggressive dose reductions and extended dosing intervals to prevent toxic delirium or cardiac complications.

### 3.2 Antipsychotic medications

The utility of antipsychotics in geriatric psychiatry is largely confined to the short-term management of severe BPSD or psychosis that poses an imminent threat to the patient or others.

#### 3.2.1 Indications and the FDA black box warning

Atypical antipsychotics (e.g., risperidone, olanzapine, quetiapine) are frequently employed off-label to mitigate agitation, aggression, and hallucinations in dementia patients (5, 34). However, this practice is heavily circumscribed by the FDA’s 2005 Black Box Warning. A meta-analysis of 17 placebo-controlled trials revealed a 1.6 to 1.7-fold increased relative risk of mortality among elderly dementia patients taking atypical antipsychotics, translating to an absolute mortality rate of 4.5% versus 2.6% in the placebo group (5, 35).

The underlying pathophysiology is multifactorial, with a significant proportion of fatalities stemming from cerebrovascular accidents (strokes), cardiac events (heart failure, arrhythmias), and infections (pneumonia) (5, 35, 36). Consequently, clinical guidelines unequivocally stipulate that APs should only be prescribed at the lowest effective dose, for the shortest possible duration, and strictly as a last resort when non-pharmacological de-escalation strategies have failed (5, 36). Given the heightened vulnerability of polymorbid geriatric patients to ventricular arrhythmias, baseline electrocardiogram (ECG) with particular attention to the corrected QT (QTc) interval is mandatory prior to initiating second-generation APs. Ongoing periodic ECG monitoring during dose titration and maintenance therapy is strongly advised to mitigate the risk of drug-induced QTc prolongation and sudden cardiac death (37). Beyond direct patient safety, appropriate management of severe BPSD reduces caregiver distress and delays nursing home placement: every 1-point reduction in Neuropsychiatric Inventory (NPI) score is associated with a 12% lower risk of caregiver burnout, making caregiver well-being a critical consideration in risk-benefit assessments of antipsychotic use.

#### 3.2.2 Second-generation APs and metabolic syndrome

While Second-Generation Antipsychotics (SGAs) are less liable to cause extrapyramidal symptoms (EPS) than their first-generation counterparts, they introduce a new set of metabolic liabilities. Olanzapine and quetiapine, in particular, are strongly associated with significant weight gain, insulin resistance, and dyslipidemia—collectively termed metabolic syndrome (38, 39).

In the geriatric population, where baseline comorbidities like Type 2 Diabetes Mellitus and hypertension are rampant, SGA-induced metabolic perturbations can be catastrophic. Routine monitoring of fasting glucose, lipid panels, and Body Mass Index (BMI) is non-negotiable. Should significant metabolic derailment occur, transitioning to a metabolically neutral agent (e.g., aripiprazole or lurasidone) is strongly advised (38, 40).

### 3.3 Emerging pharmacological targets

For patients exhibiting the neuroinflammatory phenotype or refractory to conventional monoaminergic agents (**Table 1**), novel glutamatergic and anti-inflammatory approaches are required to restore synaptic connectivity and mitigate microglial overactivation. For patients refractory to conventional monoaminergic antidepressants or those plagued by intolerable side effects, novel therapeutic avenues are actively being explored.

#### 3.3.1 Rapid-acting glutamatergic modulators (ketamine and rapastinel)

The protracted latency period (4–8 weeks) of traditional ADs is a critical drawback in acutely suicidal or severely depressed elderly patients. Ketamine, an NMDA receptor antagonist, has emerged as a paradigm-shifting agent capable of inducing rapid antidepressant effects—often within hours—by swiftly restoring synaptic connectivity through glutamate system modulation and Brain-Derived Neurotrophic Factor (BDNF) release (41, 42).

Pilot studies in Late-Life Treatment-Resistant Depression (LL-TRD) have demonstrated that repeated low-dose intravenous (IV) ketamine infusions (0.5 mg/kg) are well-tolerated in adults over 60, yielding response rates of up to 48% without precipitating severe adverse cognitive effects (43, 44). Nevertheless, stringent cardiovascular monitoring during infusions is mandatory due to transient blood pressure elevations (43). Concurrently, novel agents like Rapastinel (a GLYX-13), a NMDA receptor glycine-site functional partial agonist, are undergoing clinical evaluation, promising similar rapid efficacy with a purportedly safer side-effect profile devoid of dissociative symptoms (41).

#### 3.3.2 Anti-inflammatory agents and precision medicine

Converging evidence implicates chronic neuroinflammation as a core pathophysiological driver of LLD (45). This has catalyzed a shift towards precision psychiatry, utilizing biomarkers like C-Reactive Protein (CRP) to identify an “inflammatory phenotype” of depression. Meta-analyses indicate that anti-inflammatory agents—such as Celecoxib (a COX-2 inhibitor) or Infliximab (a TNF-α inhibitor)—exhibit significant adjunctive antidepressant efficacy, but exclusively in patients with elevated baseline inflammatory markers (e.g., CRP ≥ 2–5 mg/L) (46, 47).

Furthermore, broad-spectrum anti-inflammatories, including Omega-3 fatty acids and certain dietary interventions, have shown promise in ameliorating depressive symptoms in the elderly while concurrently mitigating age-related systemic inflammation (45, 46). Although potent biological agents (like cytokine inhibitors) carry inherent risks of immunosuppression, lifestyle-based or nutraceutical anti-inflammatory strategies represent a compelling, low-risk frontier for the precision treatment of LLD (45, 48, 49).

## 4 Psychotherapeutic interventions

Complementing biological interventions, psychotherapies for the elderly must address the HPA axis dysregulation and psychosocial deficits (**Table 1**) that perpetuate the cycle of LLD, rather than relying on standard protocols designed for younger adults. While pharmacotherapy often serves as the first line of defense in geriatric psychiatry, psychotherapy remains an indispensable pillar of comprehensive care. However, applying standard psychotherapeutic protocols to the elderly requires significant adaptation. Age-related cognitive slowing, reduced executive function, and sensory impairments necessitate “geriatric-sensitive” modifications to traditional talk therapies. Furthermore, the unique psychosocial stressors of late life—such as bereavement, chronic medical comorbidities, and caregiver dependency—demand highly pragmatic and emotionally resonant therapeutic approaches (50, 51).

### 4.1 Tailoring psychotherapies to geriatric cognitive profiles

#### 4.1.1 Modified cognitive behavioral therapy (CBT) and behavioral activation

Traditional CBT relies heavily on abstract cognitive restructuring and complex homework assignments, which can overwhelm an aging brain grappling with reduced processing speed or mild executive dysfunction (51). To bridge this gap, modified CBT for the elderly shifts the therapeutic focus away from introspective analysis and places a heavier emphasis on Behavioral Activation (BA).

BA operates on the premise that depression induces a vicious cycle of withdrawal and apathy, leading to a depletion of positive reinforcement. By collaboratively scheduling simple, mastery-oriented activities (e.g., light gardening, short walks, or social visits), therapists can help elderly patients break this cycle. Studies have shown that this pragmatic, action-oriented approach yields significant reductions in depressive symptoms while bypassing the heavy cognitive load of traditional CBT (51–53). Additionally, therapy sessions are often shortened, simplified, and supplemented with large-print written materials to accommodate visual and cognitive deficits.

#### 4.1.2 Problem-solving therapy: a pragmatic approach

Late life is frequently characterized by an accumulation of unavoidable stressors, such as the onset of chronic pain, the loss of driving privileges, or the death of a spouse. Problem-Solving Therapy (PST)​ is uniquely suited for this demographic because it is entirely present-focused and skill-based. Rather than exploring the psychodynamics of a patient’s past, PST trains older adults in a structured, step-by-step approach to navigate current life crises (54, 55).

A systematic review and meta-analysis of randomized controlled trials (RCTs) demonstrated that PST significantly outperforms control conditions in reducing major depressive symptoms in older adults, with effect sizes remaining robust at follow-up (54–57). By empowering patients with concrete tools to tackle tangible problems, PST restores a sense of self-efficacy and control—two psychological resources often eroded in the face of age-related decline.

#### 4.1.3 Reminiscence and life review therapy

Unlike younger patients who are typically oriented toward future goals, older adults often find comfort and meaning in looking backward. Reminiscence Therapy (RT)​ and Life Review Therapy (LRT)​ leverage autobiographical memory to combat LLD and despair. These therapies involve systematically discussing past experiences, either individually or in group settings, often facilitated by photographs, music, or familiar objects (58, 59).

A comprehensive meta-analysis update confirmed that LRT-REM exerts a large and significant effect in easing LLD (effect size) (58). Another meta-analysis highlighted that life review therapy significantly improved depressive symptoms in older adults with moderate to severe depression (59). By reframing past adversities, resolving lingering guilt, and integrating life achievements into a coherent personal narrative, elders can achieve a sense of “life completion” and enhanced self-worth, effectively serving as a developmentally appropriate psychotherapy for the geriatric population (58, 59).

### 4.2 Supporting the caregiving ecosystem

#### 4.2.1 Evidence-based caregiver interventions

For older adults with dementia or severe physical disabilities, the family caregiver is the backbone of the care ecosystem. However, the immense physical and emotional toll of caregiving often leads to severe burnout, depression, and premature institutionalization of the patient. Meta-analytic evidence confirms that multicomponent caregiver interventions—which combine psychoeducation, stress management, and cognitive reframing—are highly effective in reducing caregiver burden and improving their quality of life (60–63).

Furthermore, recent large-scale meta-analyses indicate that online psychosocial interventions, while showing mixed results for directly reducing caregiver burden, significantly enhance the caregiver’s sense of self-efficacy (Standardized Mean Difference [SMD] = 0.20) (63, 64). Empowering caregivers with problem-solving skills and realistic expectations not only preserves their own mental health but also drastically delays the transition of the patient into a nursing home.

### 4.3 The digital frontier: e-health and telepsychology

#### 4.3.1 Telepsychology and remote access

Geographic isolation, mobility impairments, and the stigma of visiting a psychiatrist often create formidable barriers to mental healthcare for the elderly. Telepsychology​ (the remote delivery of psychological services via video conferencing) has emerged as a powerful solution to bridge this accessibility gap.

A systematic review by Gentry et al. highlighted that telemental health (TMH) is not only highly feasible and well-accepted by older adults but also yields clinical outcomes comparable to in-person therapy for depression and anxiety (65, 66). Surprisingly, studies indicate that older adults often prefer video-based interactions over phone calls, as the visual cues help maintain engagement and reduce feelings of isolation (66). As internet infrastructure improves, integrating hybrid models of care (combining brief in-person visits with regular video check-ins) represents the most sustainable path to scaling geriatric mental health services.

## 5 Integrated care models

**Figure 3:**
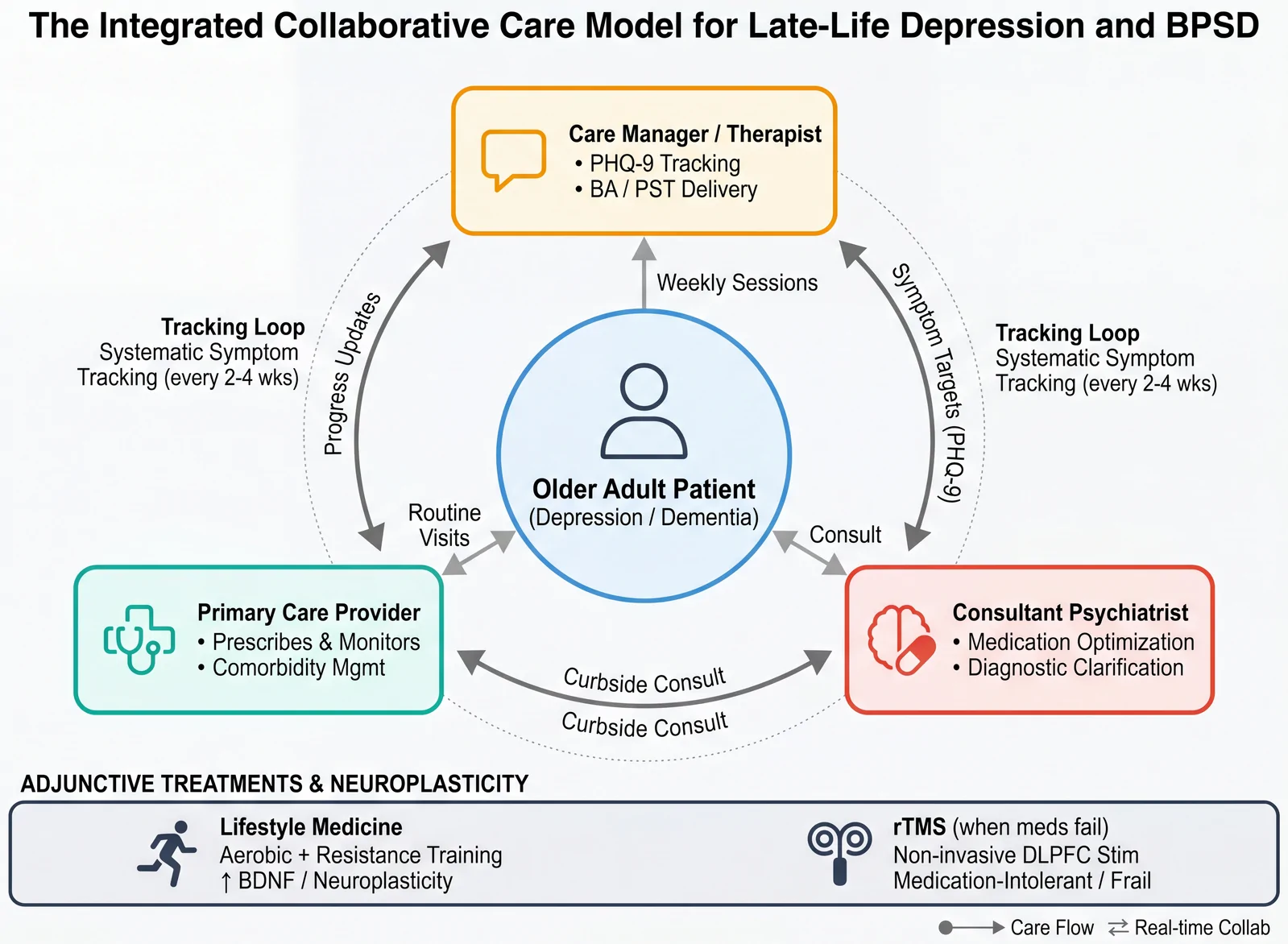
The integrated CoCM for late life.

Given the mechanistic heterogeneity outlined in **Table 1**—where vascular pathology, inflammation, and psychosocial stress intersect—fragmented single-modality care is clinically inadequate, necessitating the integrated Collaborative Care Model (CoCM). The management of geriatric mental disorders is inherently complex, often complicated by multi-morbidity, polypharmacy, and psychosocial stressors such as bereavement or caregiver burden. Consequently, fragmented, single-modality treatments are frequently inadequate. Contemporary geriatric psychiatry increasingly advocates for integrated, multidisciplinary approaches that bridge the gap between primary care, psychiatry, and allied health disciplines, as shown in **Figure 3**. **Figure 3** delineates the operational workflow of the CoCM. Unlike traditional referral systems, this model embeds a care manager within primary care to track outcomes (e.g., PHQ-9 scores) and adjust treatment plans in real-time via psychiatric consultation, ensuring continuity of care across the treatment cascade.

### 5.1 The “collaborative care” model: bridging primary care and psychiatry

Given that the vast majority of older adults with depression first seek help in primary care settings, embedding psychiatric expertise into these environments is crucial. The CoCM, pioneered by the landmark IMPACT (Improving Mood—Promoting Access to Collaborative Treatment) trial, has fundamentally reshaped the landscape of LLD management (67–69).

Unlike traditional “consultative” models where a primary care physician (PCP) merely refers a patient to a psychiatrist, CoCM establishes a tightly knit, multidisciplinary team comprising the PCP, a psychiatric consultant, and a dedicated care manager (often a nurse or clinical social worker) (67). The care manager actively tracks the patient’s progress using validated scales (e.g., PHQ-9) and facilitates evidence-based adjustments to the treatment plan in consultation with the psychiatrist (68). Robust evidence indicates that formally incorporating caregivers into the CoCM care team—including involving them in symptom tracking, treatment planning, and progress reviews—improves depression outcomes for patients while simultaneously enhancing caregiver self-efficacy, creating synergistic benefits for both patients and their support systems. Meta-analytic data demonstrate that CoCMs significantly improve depression symptom remission (OR = 1.74) and treatment response (OR = 1.78) compared to usual care, while dementia-specific CoCM programs have shown significant improvements in caregiver self-efficacy at 6 months that are largely maintained at 12 months (3, 4, 60).

The original IMPACT trial demonstrated that nearly 50% of older adults receiving CoCM achieved ≥50% reduction in depressive symptoms over 12 months versus 19% with usual care (67, 68). Subsequent economic analyses further confirmed that this integrated approach reduces acute care utilization and overall medical expenditures, establishing CoCM as both clinically effective and cost-efficient (70).

### 5.2 Lifestyle medicine: neuroprotective effects of exercise interventions

In recent years, lifestyle medicine has transitioned from a complementary approach to a core pillar of geriatric psychiatric treatment. Among various lifestyle interventions, physical exercise​ has garnered robust empirical support for its efficacy in mitigating LLD (71).

A comprehensive network meta-analysis evaluating 69 randomized controlled trials (RCTs) involving over 5,300 older adults demonstrated that aerobic, resistance (strength), and mind-body exercises (e.g., Tai Chi, Yoga) are all highly effective in reducing depressive symptoms compared to sedentary controls (71, 72). Remarkably, these modalities were found to be statistically equivalent in their antidepressant effects, suggesting that “personal preference” should dictate the chosen exercise type to maximize long-term adherence (71, 73).

The antidepressant mechanism of exercise extends beyond mere endorphin release; it actively promotes neuroplasticity. Physical activity stimulates the release of Brain-Derived Neurotrophic Factor (BDNF), which fosters hippocampal neurogenesis and synaptic connectivity—processes often impaired in the aging, depressed brain (72, 73). Furthermore, longitudinal studies, such as the renowned SMILE (Standard Medical Intervention versus Long-term Exercise) trials, have shown that while exercise is equally effective as pharmacotherapy (e.g., SSRIs) in the short term, it confers superior long-term protection against depressive relapse (74, 75). For frail older adults or those with advanced dementia, adherence to exercise regimens is heavily dependent on caregiver assistance. Brief training in motivational interviewing, goal-setting, and exercise supervision has been shown to enhance caregiver self-efficacy and confidence in supporting patients, and systematic reviews indicate that caregiver-mediated exercise interventions demonstrate potential beneficial effects on patient outcomes (3, 7, 16). Given that caregivers themselves face significant barriers to sustaining physical activity—often struggling with time constraints and competing care demands—integrating caregiver training and support into exercise prescriptions represents a critical implementation strategy for this vulnerable population.

### 5.3 Neuromodulation: rTMS for medication-intolerant patients

For a significant subset of older adults, pharmacological interventions are either ineffective or intolerable due to severe side effects, dangerous drug-drug interactions, or fragile physiological reserves. Repetitive Transcranial Magnetic Stimulation (rTMS) has emerged as a vital, evidence-based alternative for these vulnerable populations (76).

rTMS is a non-invasive neuromodulation technique that uses electromagnetic pulses to depolarize neurons in targeted cortical regions, most notably the dorsolateral prefrontal cortex (DLPFC) (76, 77). A 2023 systematic review and meta-analysis of RCTs confirmed that active rTMS is significantly more effective than sham stimulation in achieving clinical response [Odds Ratio (OR)=2.86] and remission (OR = 4.02) in patients with LLD (76).

Crucially, older adults often require higher stimulation intensities (typically ≥100% of the resting motor threshold) to overcome age-related prefrontal cortical atrophy and increased scalp-to-cortex distance (76, 77). Modern protocols utilizing deep rTMS or optimized targeting have successfully circumvented these anatomical barriers. As a result, major clinical guidelines now strongly recommend rTMS for older adults who have failed at least one adequate trial of antidepressant medication, offering a safe profile with minimal systemic side effects (77). Notably, the risk of seizure induction in older adults undergoing rTMS is extremely low (<0.1%), consistent with large-scale safety data from meta-analyses (76), further mitigating concerns about “absolute safety” often associated with non-pharmacological interventions.

## 6 Challenges, limitations, and future perspectives

**Figure 4:**
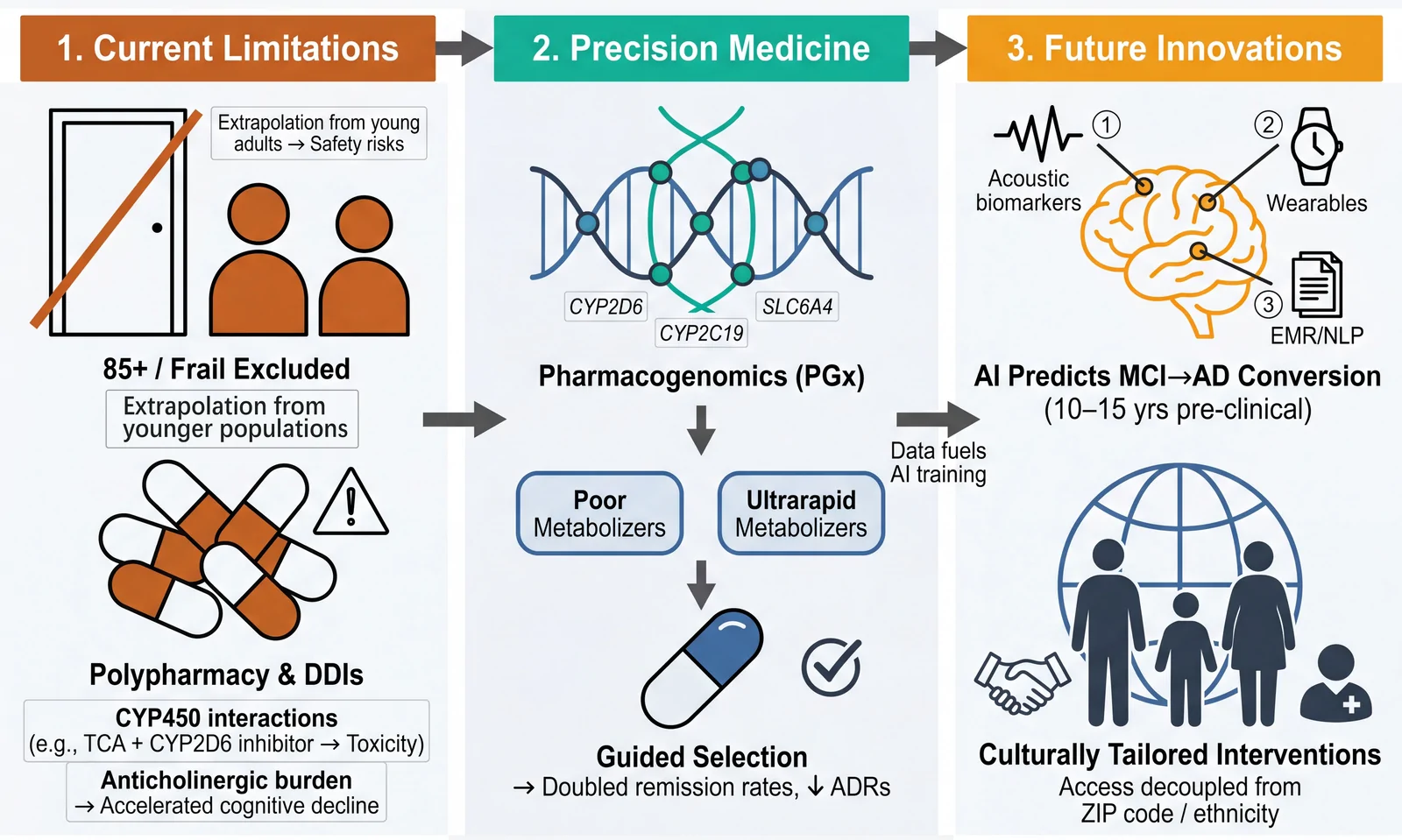
Roadmap to precision geriatric psychiatry.

Despite remarkable advancements in pharmacotherapy, psychotherapy, and neuromodulation, the field of geriatric mental health still faces significant hurdles. Transitioning from a “one-size-fits-all” approach to personalized care requires acknowledging current systemic limitations while simultaneously embracing emerging technologies and societal shifts (**Figure 4**). **Figure 4** maps the horizon of precision medicine in geriatrics, transitioning from current pharmacogenomics and AI-driven diagnostics to future equitable care frameworks. It highlights the critical pivot points where biomarker profiling and digital health technologies intersect to enable personalized treatment algorithms.

### 6.1 Current limitations in geriatric mental health care

#### 6.1.1 The “age gap” in clinical research

A persistent and troubling limitation in evidence-based geriatric psychiatry is the systematic underrepresentation of older adults in clinical trials. Regulatory analyses and systematic reviews have consistently shown that a significant proportion of clinical trials impose arbitrary upper-age limits or exclude patients based on comorbidities common in late life (e.g., cardiovascular issues, mild cognitive impairment) (78, 79).

Consequently, clinicians are often forced to extrapolate treatment guidelines from younger, healthier populations and apply them to frail, polymorbid older adults—a practice that lacks scientific rigor and poses substantial safety risks (78, 80). Bridging this “age gap” in research is not merely a methodological preference but an ethical imperative to ensure that the growing elderly population receives evidence-based care tailored to their unique physiology.

#### 6.1.2 The peril of polypharmacy and drug-drug interactions

Nowhere is the complexity of geriatric care more evident than in the management of polypharmacy—the concurrent use of five or more medications. Older adults, particularly those in long-term care facilities, frequently take multiple medications for co-existing chronic physical and psychiatric conditions (81, 82).

Psychotropic drugs, which are heavily reliant on the hepatic cytochrome P450 enzyme system for metabolism, are frequent culprits in dangerous drug-drug interactions (DDIs) (81, 83). For instance, adding a CYP2D6 inhibitor (like bupropion) to a tricyclic antidepressant (like amitriptyline) can lead to supratherapeutic levels of the antidepressant, resulting in severe toxicity and unexplained neurological symptoms (83). Furthermore, long-term polypharmacy, especially involving anticholinergic or potentially inappropriate medications, is strongly correlated with accelerated cognitive decline and a higher risk of progression to dementia (82). Recent evidence highlights that dynamic increases in anticholinergic burden (ACB) are not merely static risk factors but active drivers of multidimensional vulnerability in the hospitalized oldest-old. Elevated ACB scores correlate strongly with poorer cognitive performance (e.g., MMSE), sarcopenia, and incident delirium, with ACB remaining an independent predictor of adverse outcomes even after adjusting for comorbidities (84). This underscores the necessity of systematic medication review and deprescribing initiatives targeting high-anticholinergic-load agents to preserve functional resilience in frail geriatric populations.

### 6.2 The horizon of precision medicine

#### 6.2.1 Pharmacogenomics: decoding individual responses

To mitigate the dangers of polypharmacy and the “start low, go slow” guessing game, the field is rapidly turning toward pharmacogenomics. PGx testing analyzes clinically important genetic variations—such as polymorphisms in the *CYP2D6*, *CYP2C19*, and serotonin transporter (*SLC6A4*) genes—that dictate how an individual metabolizes psychotropic medications (85, 86).

Recent randomized controlled trials (RCTs) have demonstrated that combinatorial pharmacogenomic testing significantly improves outcomes for older adults with major depressive disorder. By guiding medication selection to avoid gene-drug interactions, PGx testing has been shown to double the rate at which patients are placed on optimal medications, thereby increasing remission rates and reducing adverse drug events (85, 86). Integrating PGx into routine geriatric psychiatric practice promises a future where medication selection is driven by an individual’s unique genetic blueprint rather than trial and error.

#### 6.2.2 Implementation challenges of precision medicine

Despite the transformative potential of these precision-guided approaches, several pragmatic barriers impede its widespread adoption in geriatric psychiatry. First, accessibility and reimbursement remain major hurdles.​ While pharmacogenomic (PGx) testing is commercially available, its uptake in routine geriatric care remains low due to inconsistent insurance coverage and high out-of-pocket costs (87). Second, ethical and regulatory complexities surround emerging technologies.​ AI-driven diagnostic tools often exhibit “algorithmic bias,” as training datasets predominantly feature Caucasian populations, potentially leading to misdiagnosis in ethnic minorities. Furthermore, the “black box” nature of deep learning models complicates clinician accountability and informed consent processes (88, 89). Third, workforce readiness is a critical bottleneck.​ A global shortage of geriatric psychiatrists means primary care physicians—who manage the majority of older adults—often lack the specialized training to interpret PGx results or integrate biomarker data into complex polypharmacy regimens (90). Finally, cost-effectiveness data specific to older adults are scarce.​ While PGx testing may reduce long-term medication costs, the upfront investment and lack of robust health economic models tailored to frail elderly populations limit stakeholder buy-in (91). Addressing these multidimensional challenges requires multi-stakeholder collaboration to standardize clinical guidelines, secure sustainable funding, and develop user-friendly clinical decision support tools that demystify precision medicine for the frontline clinician.

### 6.3 Future directions and innovations

#### 6.3.1 Artificial intelligence (AI) in early detection

The future of geriatric mental health lies heavily in predictive analytics and Artificial Intelligence. LLD and neurocognitive disorders (like Alzheimer’s) often share overlapping symptoms, making early differential diagnosis exceedingly difficult. AI algorithms, particularly those utilizing Natural Language Processing (NLP) and machine learning, are proving to be revolutionary.

For example, novel AI tools can now analyze subtle acoustic features in a patient’s spontaneous speech or track passive behavioral data via wearable sensors to detect cognitive decline and predict the conversion from mild cognitive impairment to dementia up to 10–15 years before clinical onset, with astonishing accuracy (92). Complementing these behavioral signatures, AI-driven neuroimaging analysis is adding a deeper layer of biological granularity. Deep learning algorithms, particularly convolutional neural networks (CNNs), can automatically quantify subtle structural changes—such as cerebral microbleeds, white matter hyperintensity volumes, and hippocampal atrophy—from routine CT and MRI scans (93, 94), often surpassing human expert performance in predicting MCI-to-dementia conversion (93, 95). Furthermore, the emergence of radiomics allows for the extraction of high-dimensional features from imaging voxels (94), enabling the construction of ‘imaging-genomics’ models that stratify patients into biologically distinct subtypes (96, 97). This integration of macro-scale imaging with micro-scale molecular data is pivotal for realizing the precision medicine roadmap outlined in **Figure 4**. Such tools allow for unprecedented early intervention, potentially altering the trajectory of neurodegenerative diseases before irreversible damage occurs.

#### 6.3.2 Global health disparities and equitable implementation

The global landscape of geriatric mental health is marked by profound inequities. According to the Global Burden of Disease Study 2021, LLD burden is disproportionately higher in low sociodemographic index (SDI) regions—particularly Central and Eastern Sub-Saharan Africa—compared to high-SDI regions, with disparities in healthcare infrastructure and policy serving as key drivers (98). The World Health Organization (WHO) Global Dementia Observatory reveals a stark disparity​ in the density of geriatric psychiatry specialists (including geriatricians and old-age psychiatrists) between low- and middle-income countries (LMICs) and high-income countries—a gap exceeding 100-fold. Furthermore, according to the WHO Mental Health Atlas 2024, governments globally allocate a median of only 2.1% of total health budgets​ to mental health, with public spending ranging from 0.6% to 4.3% across 75 surveyed countries, and even lower in LMICs. This severe underinvestment leaves the vast majority of mental health cases in low-resource settings untreated (99).

Cultural stigma represents another formidable barrier. In many Eastern societies and LMICs, mental illness frequently encounters social prejudice, leading to treatment delay or avoidance, while somatic masking of depressive symptoms further complicates recognition. These cultural dynamics, coupled with workforce shortages, necessitate adaptive implementation strategies.

Encouragingly, localized adaptations of the CoCM have demonstrated feasibility and efficacy across diverse settings. In urban China, a cluster-randomized trial of Depression Care Management (DCM) achieved a Cohen’s d of 0.8 in reducing Hamilton Depression Rating Scale scores among adults ≥60 years over 12 months (100). The rural China COACH trial further confirmed that CoCM delivered by village doctors and aging workers, under psychiatrist consultation, significantly improved both depressive symptoms and hypertension control. In India, the MANAS trial pioneered “task-shifting” by deploying lay health counselors to deliver collaborative stepped-care, substantially reducing the prevalence of common mental disorders (101). Similarly, a real-world cohort study in South Africa validated task-sharing CoCM with co-located counseling in underserved primary healthcare settings (102).

These exemplars underscore the viability of task-shifting strategies—delegating mental health screening, basic psychosocial interventions, and care coordination to community health workers, nurses, and lay counselors under specialist supervision. The WHO and the World Psychiatric Association have explicitly endorsed task-shifting as a scalable solution for LMICs. In these resource-constrained settings, family caregivers serve as the de facto frontline providers of geriatric mental healthcare: supplementing task-shifting training for community health workers with brief, culturally tailored caregiver education on symptom recognition, de-escalation techniques for BPSD, and self-care strategies is essential to sustain treatment gains and prevent caregiver burnout in the absence of specialist support. Looking forward, achieving genuine equity in geriatric mental health demands more than translation of evidence-based protocols; it requires deep cultural adaptation, integration with primary healthcare platforms, sustainable funding mechanisms, and deliberate investment in local workforce development.

## 7 Conclusion

The demographic trajectory of global aging necessitates a paradigm shift in mental health care—one that moves decisively away from the traditional, albeit outdated, “one-size-fits-all” approach. As demonstrated throughout this review, the complexities of multimorbidity, polypharmacy, and age-related physiological changes demand a nuanced, multidisciplinary, and highly individualized model of care.

A cornerstone of modern geriatric psychiatry is the recognition that older adults are not merely “smaller adults.” Age-related pharmacokinetic shifts—including reduced hepatic clearance and declining renal function—necessitate the long-standing “start low, go slow” dosing principle, coupled with vigilant monitoring for adverse drug reactions and drug-drug interactions. Equally critical is the systematic integration of caregiver support into all levels of geriatric mental healthcare: routine assessment of caregiver burden, provision of evidence-based caregiver psychoeducation, and inclusion of caregiver-reported outcomes in treatment response evaluations must become standard practice, as sustainable improvements in patient outcomes are inextricably linked to the well-being of their care ecosystems. Future pharmacotherapy must prioritize safety, favoring agents with minimal anticholinergic burden to prevent iatrogenic complications such as falls, delirium, and cognitive decline. Ultimately, the future of geriatric psychiatry lies in the implementation of mechanism-guided precision care (**Table 1**). Moving beyond syndromic diagnosis, clinicians must assess and target the specific pathophysiological drivers—be it vascular, inflammatory, or neurodegenerative—to optimize outcomes for this vulnerable population.

Similarly, psychological interventions cannot be applied to the elderly without thoughtful adaptation. Age-specific psychological therapies must account for the unique psychosocial challenges of late life, including bereavement, retirement, chronic pain, and functional dependency. Effective psychotherapy in this demographic requires cultural sensitivity and linguistic accessibility. Therefore, the localization and cultural adaptation of evidence-based therapies (such as CBT, Problem-Solving Therapy, and Reminiscence Therapy) are essential to ensure therapeutic alliance, enhance patient engagement, and address the specific existential concerns of diverse elderly populations.

Persistent gaps in the evidence base—particularly the underrepresentation of adults aged ≥85 years and medically frail individuals in RCTs—limit the generalizability of current treatment guidelines. Future research must prioritize pragmatic trial designs that enroll these historically excluded subgroups to generate real-world applicable evidence, ensuring equitable access to safe, effective, and dignified mental healthcare for all older adults.
